# Supplementary material for: Perspectives of health and community stakeholders on community-delivered models of malaria elimination in Lao People’s Democratic Republic: A qualitative study
Source: PLoS One. 2022 Mar 10;17(3):e0264399. doi: 10.1371/journal.pone.0264399 (PMC8912149; doi:10.1371/journal.pone.0264399)
Supplement: S1 File — (DOCX) [file pone.0264399.s001.docx]

## **Supporting information file 1. Services provided by village health volunteers (VHVs) and village malaria workers (VMWs)**

| **Services** | **VHV** | **VMW** |
| --- | --- | --- |
| ***Malaria-specific health services*** |  |  |
| Provide rapid diagnostic test | **√** | **√** |
| Provide antimalarial treatment for uncomplicated malaria | **√** | **√** |
| Refer complicated malaria and malaria with pregnancy patients to nearest health facility | **√** | **√** |
| Organise the distribution of insecticide-treated bed nets | **√** | **√** |
| Provide health education related to malaria prevention | **√** | **√** |
| Serve as first-tier malaria surveillance units | **√** | **√** |
| Compile and report village-level malaria data to health centres | **√** | **√** |
| ***Other general health services*** |  |  |
| Assist health centre staff in outreach activities | **√** |  |
| Provide health education and community health promotion services | **√** |  |
| Provide basic health care using first aid kit | **√** |  |
| Facilitate antenatal care | **√** |  |
| Vital event surveillance | **√** |  |
| Patient referral | **√** |  |
