# Supplementary material for: Perspectives of health and community stakeholders on community-delivered models of malaria elimination in Lao People’s Democratic Republic: A qualitative study
Source: PLoS One. 2022 Mar 10;17(3):e0264399. doi: 10.1371/journal.pone.0264399 (PMC8912149; doi:10.1371/journal.pone.0264399)
Supplement: S2 File — (DOCX) [file pone.0264399.s002.docx]

## **Supporting information file 2. Topic guides for focus group discussions and interviews**

**Interview topic guide for Key Informant Interviews (KII) and In-depth interviews (IDI)**

Semi-structured interview

- Introduction and ice breaking exercise
- Summary presentation of the study to the participants
- Non-identifiable information explained to the participants
  - Record the age, sex, organization in which she/he works, position/level in the organization without identifying the duty station, roles and responsibilities in summary

Draft themes, questions and probes

- **Current malaria situation and control measures**
  - Can you please tell me about the malaria situation in Lao PDR?
  - Why do you consider that the malaria trends demonstrate that there is the change in the malaria prevalence in LaoPDR?
  - What’s the situation of malaria in your assigned territory?
  - Why do you think that the burden of malaria in your area is going up/down or plateauing?
  - What are the current malaria control interventions and models in your area?
  - What are the relative contributions of each model to the success of malaria control? How do you rate them and why?
  - Who are the key stakeholders in successful malaria control in your area?

- **Views and perspectives on the current malaria VHV model**
  - How do you think that the VHVs contribute to malaria control in Lao PDR?
  - Probe: Importance of their role in malaria control in Lao PDR?
- **Policy and strategic barriers and enablers for Lao malaria elimination (to omit this theme in IDI)**
  - What are the policies, strategies or regulations that have led to the malaria elimination approach in Lao PDR? How do these policies, strategies and/or strategies play a role in malaria elimination?
  - What policies, strategies and regulations hinder malaria elimination in Lao PDR? How do these policies, strategies and regulations cause hindrances to malaria elimination?
- **Operational barriers and enablers for Lao malaria control and elimination using community delivered models**
  - Which approaches worked and which didn’t in applying community delivered models in the field? Why did this occur?
  - What are the operational barriers and enablers in managing the community volunteers and the model?
  - How do the barriers and enablers impact in the success or failure of the malaria control and elimination model? Why?
- **Strategies to maintain the motivation and social role of VHVs in the community**
  - How do you think the motivation and social role of VHVs in the community affects malaria control and elimination?
  - How do we maintain VHVs’ motivation and social role in the community?
  - What are the rationales for your suggestions?
- **The factors need to be addressed during the transition from malaria control to elimination using community delivered models**
  - In the future, what role do you think the VHVs can play in the malaria elimination model? Why?
  - How can we adapt the existing role of VHVs in malaria control to fit into the proposed elimination model?
  - Which aspect of current malaria control model do you think can be strengthened in the future? In order to do so, what resources are needed?

- **Additional topic identified by the interviewee**
  - Is there anything you like to add or discuss? Please discuss more.

End of the interview

**Participatory workshop guide**

**Responsible persons**

Main Facilitator (MF) from LaoTPHI – lead the overall process and facilitate to collect enriched data using an ethical approach

Additional facilitator 1 (F1) – Note taking, audio recording and supplementary facilitation

Additional facilitator 2 (F2) (optional) – logistic, administrative, financial managements and supplementary facilitation, translation (if facilitator 2 is not involved, main facilitator and facilitator 1 will share the responsibilities of facilitator 2)

**Agenda**

| **No** | **Session** | **Time** | | **Facilitator (F)** |
| --- | --- | --- | --- | --- |
| 1 | Introduction and informed consent | 8:00 | 8:20 | MF |
| 2 | Introduction of malaria, situation and interventions of malaria in | 8:20 | 8:50 | MF |
| 3 | Theme 1 small groups discussion (preference ranking or matrix scoring) applying questions and probes and 5 minutes presentations | 8:50 | 9:50 | MF, F1 and F2 |
| 4 | Theme 2 small groups discussion (diagramming and visualization – social maps) applying questions and probes and 5 minutes presentations |  |  |  |
| 5 | Theme 3 small groups discussion (semi-structure interview) applying questions and probes and 5 minutes presentations |  |  |  |
|  | Tea break | 9:50 | 10:00 | All |
|  | Theme 4 small groups discussion (diagramming and visualization – social maps) applying questions and probes and 5 minutes presentations | 10:00 | 11:00 | MF, F1 and F2 |
|  | Theme 5 small groups discussion (diagramming and visualization – resource maps) applying questions and probes and 5 minutes presentations |  |  |  |
|  | Theme 6 small groups discussion (preference ranking) applying questions and probes and 5 minutes presentations |  |  |  |
|  | Theme 7 small groups discussion (diagramming and visualization – mind maps) applying questions and probes and 5 minutes presentations | 11:00 | 11:40 | MF, F1 and F2 |
|  | Conclusion and group recommendations | 11:40 | 12:00 |  |
|  | Lunch break | 12:00 | 13:00 |  |

**Theme, questions and probes**

| **No** | **Themes** | **Questions and probes** |
| --- | --- | --- |
| 1 | Current malaria situation and priority health problems in the respective community | - Has your village/community ever experienced malaria? - If yes, how did it impact on your village/community? - How did you manage to help the patients? - Do you think malaria is still a priority health issue in your village/community   - - Why do you think so? - What are the other health problems in your village/community?   - - How do you rank them? |
| 2 | Malaria control measures and available health services in the respective community | - What are the sources of health care services for your community?   - - Which sources do you prefer and why?     - Which sources do you not prefer and why? - What are the available services/interventions for malaria in your community?   - - Do you think are they enough to compact malaria?     - If not, what interventions are you going to suggest? - Where does your community get the malaria interventions/services recently?   - - Do you think is it suitable?     - If not, why and what’s your suggestion to meet the needs? |
| 3 | Views and perspectives on the current malaria VHV models | - Have you ever received services from the VHV in your village/community?   - - If yes, tell me your story/experience     - If no, can you tell me other people’s experiences in your village? - Do you think current VHV in your village/community is effective to control malaria?   - - Why? And How? |
| 4 | Policy and strategic barriers and enablers for effective malaria control and elimination in their community | - Have you ever noticed that the local policy, rules and regulations caused barriers and/or enablers for the malaria interventions in the community?   - - What are they?     - How did they affect the interventions     - How do we enhance the enablers in the future?     - How do we overcome the barriers in the future? |
| 5 | Available community supports for malaria control and elimination in their community | - Did the community support the VHV in the past for malaria activities?   - - How did they support? - Do you think the VHV should get local supports from the community in the future?   - If yes, why?     - What supports they should receive to effectively work for the community?     - How can the community support them?   - If no, why?     - Do you think they will survive without community support? |
| 6 | Strategies to maintain the motivation and social role of VHVs in the community | - What is your opinion on health care and social role of the VHV in your village/community?   - - Was it declining or increasing compared to the past 2-3 years?     - Why do you think it was happening so?     - How has the VHV responded it? - Do you think is it necessary to maintain/uplift the role of VHV in the village/community? - How do we maintain them?   - - Can you elaborate each strategy in detail? |
| 7 | Culture, customs and norms of the ethnic communities that play as barriers and enablers for effective malaria control and elimination in their community | - Have you ever noticed that the local culture, customs and norms of the ethnic communities caused barriers and/or enablers for the malaria interventions in the community?   - - What are they?     - How did they affect the interventions?     - How do we enhance the enablers in the future?     - How do we overcome the barriers in the future? |

**Focus Group Discussion (FGD) guide**

**Introduction**

Now that I have explained the purpose of our study and you have all agreed to participate, can we begin the discussion?

Theme, questions and probes

| **No** | **Themes** | **Questions and probes** |
| --- | --- | --- |
| 1 | Current malaria situation and priority health problems in your community | - Do you know what malaria is?   - - Have you or your family ever experienced malaria?     - If yes, how was it impacted on you / your family?     - If no, have you ever heard of anyone in your village/community suffered from malaria? - Do you think malaria is still a priority health issue in your village/community   - - Why do you think so? - What are the other health problems in your village/community   - - How do you rank them? |
| 2 | Malaria control measures and available health services in the respective community | - What are the sources of health care services for your community?   - - Which sources do you prefer and why?     - Which sources do you not prefer and why? - What are the available services/interventions for malaria in this community?   - - Do you use them, when? How have they worked?     - What do you think about the current malaria services in your community? |
| 3 | Views and perspectives on the current malaria VHV models | - Have you ever received services from the VHV in your village/community?   - - If yes, tell me your story/experience     - If no, can you tell me other people’s experiences around you? |
| 4 | Available community supports for malaria control and elimination in their community | - What supports did the VHV receive from the community for malaria activities?   - - How did the community support? - Can you explore the reasons for the VHVs why they should or shouldn’t get local supports from the community?   - - What supports should they receive to effectively work for the community?     - How can the community support them?     - If you think the community shouldn’t support them, why? |
| 5 | Strategies to maintain the motivation and social role of VHVs in the community | - What is your opinion on health care and social role of the VHV in your village/community?   - - Was it declining or increasing compared to the past 2-3 years?     - Why do you think it was happening so?     - How has the VHV responded to it? - What is your opinion on maintaining/uplifting the role of VHV in the village/community? - How do we maintain them?   - - Can you elaborate each strategy in detail? |
| 6 | Culture, customs and norms of the ethnic communities that play as barriers and enablers for effective malaria control and elimination in their community | - Are there any barriers or enablers to people in the community accessing the malaria interventions?   - - What are they? Local customs, experiences?     - How did they affect the interventions?     - How do we enhance the enablers in the future?     - How do we overcome the barriers in the future? |

This is the end of my questions. Do you have anything else you would like to say about this project?

Do you have any questions for me?

Thank you very much for participating.

**End of session**
